# Association maladie cœliaque et tuberculose multifocale: à propos d’un cas avec revue de la littérature

**DOI:** 10.11604/pamj.2017.27.214.12847

**Published:** 2017-07-20

**Authors:** Khadija Sarghini, Sofia Oubaha, Zouhour Samlani, Khadija Krati

**Affiliations:** 1 Service de Gastroentérologie, Centre Hospitalier Universitaire Mohamed VI de Marrakech, Marrakech, Maroc; 2 Laboratoire de Physiologie, Faculté de Médecine et de Pharmacie de Marrakech, Marrakech, Maroc

**Keywords:** Maladie cœliaque, tuberculose, association, augmentation du risque, Coeliac disease, tuberculosis, association, increased risk

## Abstract

La maladie cœliaque est une entéropathie auto-immune liée à l'intolérance au Gluten qui survient sur un terrain génétiquement prédisposé. Le diagnostic repose sur la combinaison d'arguments cliniques, biologiques et histologiques. Elle est associée à de nombreuses complications, notamment le lymphome. Le risque de tuberculose chez les patients cœliaques est augmenté. Plusieurs hypothèses expliquant cette association ont été discutées. Nous rapportons un cas de maladie cœliaque associée à la tuberculose multifocale chez une patiente âgée de 17 ans.

## Abstract (en)

Coeliac disease is an autoimmune enteropathy related to gluten intolerance which occurs in subjects genetically disposed. The diagnosis is based on the combination of clinical biological and histological arguments. It is associated with many complications, including lymphoma. The risk of tuberculosis in patients with coeliac disease is increased. Several hypotheses explaining this association have been discussed. We report a case of coeliac disease associated with multifocal tuberculosis in a 17-year old patient.

## Introduction

La maladie cœliaque est une maladie dysimmunitaire systémique, liée à l'intolérance au Gluten, survenant chez des sujets génétiquement prédisposés, et caractérisée par la combinaison variable de manifestations cliniques diverses, d'anticorps spécifiques et d'une entéropathie chez les personnes ayant le phénotype HLA DQ2 ou DQ8 [1]. Elle est souvent caractérisée par la dénutrition et liée à un certain nombre de complications, comme le lymphome [2]. La tuberculose reste un problème de santé publique dans notre contexte [3], favorisé par plusieurs facteurs dont la dénutrition. Plusieurs études et cas cliniques ont rapporté cette association entre la maladie cœliaque et la tuberculose [4]. Son risque est augmenté chez les cœliaques. Nous rapportons un cas de maladie cœliaque associée à la tuberculose multifocale chez une patiente âgée de 17 ans.

## Patient et observation

**Figure 1:**
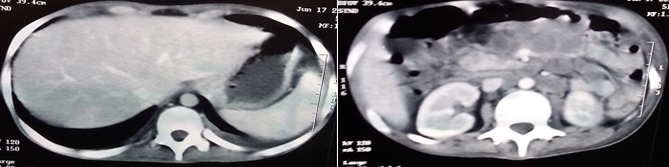
TDM thoraco abdominale montrant des adénopathies médiastinales, intra-et rétro-péritonéales avec atteinte broncho pulmonaire, un épanchement pleural minime et péritonéal avec un épaississement iléo caecal

Il s'agit d'une patiente âgée de 17 ans, sans antécédents pathologiques particuliers, admise pour bilan d'ascite associée à des douleurs de la fosse iliaque droite et une aménorrhée secondaire évoluant dans un contexte d'altération de l'état général. L'examen clinique a objectivé des adénopathies cervicales et des signes de dénutrition. Le bilan a mis en évidence une ascite riche en protide avec un taux d'adénosine désaminase dans le liquide d'ascite à 40 U/l. L'échographie abdominale a retrouvé un épaississement du carrefour iléocæcal associé à une infiltration de la graisse mésentérique et des coulées d'adénopathies profondes. Le bilan de tuberculose a montré une intradermo réaction à la tuberculine négative, des BK crachats négatifs et à la radiographie du thorax un aspect d'élargissement médiastinal et de scissurite. Le bilan biologique a objectivé des signes de malabsorption, le bilan endoscopique a mis en évidence une pangastrite érythémateuse à l'endoscopie digestive haute avec réalisation de biopsie duodénale et un aspect pseudopolypoide ulcéré et rétracté de la région iléo caecale à la coloscopie. La TDM thoraco abdominale (Figure 1) a révélé des adénopathies médiastinales intra-et rétro-péritonéales avec une atteinte broncho pulmonaire, un épanchement pleural minime et péritonéal avec un épaississement iléo caecal. Le tableau clinique plaidait en faveur de tuberculose multifocale pleurale, pulmonaire, ganglionnaire, iléo-caecale et péritonéale, et c'est la biopsie des ganglions cervicaux qui a permis de confirmer le diagnostic en montrant une tuberculose caséofolliculaire, avec une sérologie HIV négative. Alors que la biopsie duodénale était en faveur d'une maladie cœliaque sous jacente en montrant une atrophie villositaire stade 3. La patiente fut mise sous régime sans gluten associé au traitement anti bacillaire. L'évolution était marquée par l'amélioration clinique, biologique et morphologique.

## Discussion

La prévalence de la maladie cœliaque se situe entre 1/2500 et 1/3 000 pour les formes symptomatiques classiques, mais la majorité des formes sont silencieuses, ont une symptomatologie atypique et sont souvent méconnues [5]. Dans les pays occidentaux, la prévalence se situe entre 0.7 et 2% dans la population générale [5]. La fréquence varie selon l'origine ethnique, des incidences proches de celles de l'Europe ou des États-Unis sont notées en Afrique du Nord, au Moyen-Orient ou en Inde. En revanche, la maladie cœliaque est quasiment inconnue en Asie du Sud Est et en Afrique noire. Son tableau clinique est polymorphe. Elle est progressivement passée du statut de maladie digestive rare du nourrisson à celui de maladie systémique fréquente touchant tous les âges de la vie. C'est une entéropathie chronique avec atrophie villositaire secondaire à une réponse immunitaire inappropriée de la muqueuse intestinale à la gliadine du blé, de l'orge et du seigle. Ce dernier n'est toxique que chez des sujets génétiquement prédisposés [6]. Les séquences peptidiques toxiques de la gliadine sont relativement résistantes aux capacités enzymatiques digestives et peuvent parvenir intactes au contact de la muqueuse intestinale. Ces fragments sont alors absorbés par l'épithélium et arrivent dans le chorion au contact de la transglutaminase tissulaire dont ils sont des substrats de par leur richesse en glutamine. La transglutaminase transforme par désamidation, les glutamines chargées positivement en résidus d'acides glutamiques, chargés négativement. Ceci permet alors leur liaison aux poches à peptides, chargées positivement, des molécules HLA DQ2 ou DQ8 qui sont situées à la surface des cellules présentatrices d'antigènes. Ces peptides désamidés sont reconnus par les lymphocytes T CD4+ intestinaux qui produisent alors des cytokines comme l'interféron, l'IL 4 et le TNF, responsables des lésions d'inflammation et d'atrophie villositaire [7]. La tuberculose reste à nos jours un problème de santé publique dans les pays en voie de développement et notamment dans le Maroc [3]. Les mouvements migratoires, l'utilisation de plus en plus fréquente de thérapeutiques immunosuppressives et l'infection par le virus D'immunodéficience humaine (VIH) sont responsables de la recrudescence de la tuberculose dans les pays industrialisés. Environ un tiers de la population mondiale est infecté par Mycobacterium tuberculosis, mais la tuberculose active se produit chez 5 à 10% des personnes exposées [8].

La localisation pulmonaire est la plus fréquente. Le premier lien entre maladie cœliaque et tuberculose a été suggéré par Williams en 1952 [9]. Par la suite, plusieurs cas cliniques et études ont conforté cette association [10]. Dans une étude britannique, six des 76 patients adultes atteints de maladie cœliaque ont rapporté une histoire de tuberculose et sept autres avaient des preuves radiologiques de la tuberculose [11]. Cependant, la plupart des recherches se limitent à des petites séries et des rapports de cas. En 2007, le groupe de recherche de Ludvigsson et al a étudié le risque de tuberculose chez 14 335 personnes atteintes de maladie cœliaque dans une étude de cohorte suédoise, et a constaté que ces patients cœliaques ont un risque élevé, plus de 4 fois que la population générale, de développer la tuberculose [12 ]. Cette étude avait beaucoup de limites [12], ce qui a poussé ce même groupe a mené une autre étude en 2010. Cette fois-ci, ils ont conclu à un risque de tuberculose à 2(HR = 2.0) et le risque absolu était de 10/100 000 années-personnes [13]. Plusieurs hypothèses expliquant cette association ont été discutées. On a montré que la vitamine D induit la synthèse de monoxyde d'azotes au niveau des macrophages, entraînant la destruction de M. tuberculosis [14]. La maladie cœliaque peut entraîner une mauvaise absorption de la vitamine D et son faible taux sérique est associé à un risque augmenté de tuberculose active, comme le montre une méta-analyse récente d'études antérieures [15]. D'autres mécanismes ont été récemment élucidés, y compis le rôle de la vitamine D avec IL-15 pour méditer l´effet de stimulation induite par les agents pathogènes et la reconnaissance du Récepteur TLR2 pour induire l'expression de l'antimicrobien dans les macrophages [16]. Une fréquence augmentée de CD25+ et des cellules T régulatrices a été décrite chez les patients cœliaques [17]. L'augmentation de la fréquence des cellules T reg atténue la réponse des cellules T effectrices nécessaire pour combattre M. tuberculosis avec un risque plus élevé à développer la tuberculose patente [18]. L'intérêt de ces cellules dans ce sens est en cours d'étude [19]. La maladie cœliaque est une situation d'inflammation chronique, associée à une augmentation concomitante de l'expression de CTLA-4 et PD-1 sur les cellules immunitaires, entrainant une baisse de la réponse des cellules Th17 and Th1; et donc responsable d'une diminution de la réponse immunitaire. Cette situation facilite l'infection par M.tuberculosis vue la baisse de la réponse immunitaire, notamment Th1 [20].

## Conclusion

Selon les données de littérature, la maladie cœliaque augmente le risque de tuberculose, ce qui peut retarder le diagnostic si elle est révélatrice d'où l'intérêt des biopsies duodénales au moindre doute.

## Conflits d’intérêts

Les auteurs ne déclarent aucun conflit d'intérêts.
